# Supplementary material for: The evolution of scientific literature as metastable knowledge states
Source: PLoS One. 2023 Jul 12;18(7):e0287226. doi: 10.1371/journal.pone.0287226 (PMC10337867; doi:10.1371/journal.pone.0287226)
Supplement: S1 Appendix — (PDF) [file pone.0287226.s001.pdf]

S1 Appendix.

Parameter selection with an initial run of logistic regression

We perform multivariate logistic regression to evaluate significance of endogenous variables to knowledge evolution, and to select parsimonious inputs for our predictive model. We use a simplified version of purposeful selection [1] to avoid overfitting and overdependence on observed data. Here we present detailed results of the initial run. Among the statistically insignificant variables, note that network interdisciplinarity's marginal effect was significant, so its presence was worth evaluating in a more parsimonious second model. Reported in our main results, this second model, confirmed the significance of network interdisciplinarity. We therefore dropped the other insignificant variables before our predictive model. We do note that pseudo-R^2 went down slightly (0.1025 vs. 0.1109), so future work should investigate the as-yet insignificant explanatory power that the removed exogenous variables provide as part of a wider effort to understand and explain knowledge metastability.

Parameter selection with grid search

For tuning the hyperparameters (maximum tree depth, number of tress) for the random forest model, we performed a coarse grid search with search space consisting of maximum tree depth value from 3 to 15 and number of trees ranging from 20 to 100 in increments of 5. The results showed that the highest values of F1 score are achieved in the range of 35 to 50. Based on the observations, we performed a finer search for number of classifiers between 35 to 50 by incriminating the value by 1. A sample of the results from the finer search are shown in Figure in S2 Figure.

Experiments with varying train/test split

In order to understand the effects of size of training data on model performance, we explore the model parameter space (number of classifiers, maximum depth of each classifier) for different train/test splits starting with training on 2011-14 and predicting on 2015. We subsequently increase the size of training data set by adding events occurring in the following years in one year increments and predict on the subsequent year. Best F1 score for each train/test split is given in Figure in S1 Figure. Performance generally increases with training data set size, although overfitting occurs in two of our experiments.

Experiments varying cosine similarity threshold

To better understand the impact of threshold choice for cluster matching, we have carried out a series of experiments with varying thresholds. As one would expect, increasing the threshold (tightening the criteria for a cluster at time t to be considered the same as a cluster at time $t + 1$) leads to a decrease in the number of identified merges and splits. And consequently, an increase in the number of continuation and death events. Threshold values above 0.97 result in no merge events while lowering the threshold below 0.88 results in no continuation events. Figure in S3 Figure, and Figure in S4 Figure gives a summary of impacts of threshold choice on observed events. In this way, we

can interpret the cluster similarity threshold as a parameter modulating selection from amongst a *hierarchical family of events* varying in resolution.

With respect to prediction task performance, performance is relatively stable for values between 0.88 and 0.91. However, increasing the threshold above 0.91 led to reduction in the performance in our dataset. We hypothesize this may be due to the reduction in number of events identified and thus less training.

Expert feedback

We solicited feedback from three domain experts - one each from the fields of Marketing, Psychology, and Political Science. Specifically, we asked them to complete two tasks.
Task 1: We showed each expert titles and abstracts corresponding with a collection of publications in their field (13 marketing papers, 12 each for psychology and political science). We asked them to group those papers into a fixed set of clusters (4 for marketing, 3 for psychology and political science).
Task 2: After they had completed their own clustering, we showed them the clusters our algorithm identified. We asked them to rate the clusters using a Likert scale (1 to 5, where 1 represents *extremely bad* and 5 represents *extremely good*).
Results are summarized in Table in S2 Table. We use Jaccard similarity to score similarity between the expert-generated clusters and our clusters (Task 1). Despite variation in this outcome across the three fields, experts consistently gave high ratings when evaluating the clusters created by our model (Task 2). This appears to highlight the inherent challenge of coming up with a universal "ground truth" for defining similar papers or groups of papers in the literature.
Our survey also included an open field for any additional feedback. Following is the feedback we received:

Marketing: *One paper (13) is pure theory and more Econ but the others are data driven marketing papers*
Psychology: *The B category hangs well together with health and intervention work. It is the A and C groups that are more challenging as they cut across social and cognitive sciences. e.g., paper 6 is about collective cognition which should likely be paired with paper 12*
Political Science: *This was perfect – but imo this one was pretty easy, I could do the clustering through the titles alone*

Of note, the faculty member from Psychology defined clusters based on normative subfields within the discipline. Conversely, the faculty member from Marketing used differences in the theoretical vs. applied focus of the work as a primary criteria. These anecdotes offer insight into why previous work in science of science has generally relied on subject categories generated by sources like Web of Science as ground truth. However, we argue that existing categories are very high level and do not adequately account for the interdisciplinary nature of much of the literature.

Feature ablation study

To further understand the individual contributions of the language and network feature sets to model performance, we conduct ablation studies. First, the model is trained with only lingustic features (number of strong, number of weak members, and mean language ID score); grid search was performed to find optimal hyperparameters. This approach achieved **F1 0.72** on the hold-out dataset. Similarly, we train and test the model using only network features *mean network ID for strong, and weak members.* This approach

results in overfitted models with best **F1 0.581**. Finally, the model trained on features selected using purposeful selection achieves **F1 0.814**.

References

1. Hosmer Jr DW, Lemeshow S, Sturdivant RX. Applied logistic regression. vol. 398. John Wiley & Sons; 2013.
