# Supplementary material for: The evolution of scientific literature as metastable knowledge states
Source: PLoS One. 2023 Jul 12;18(7):e0287226. doi: 10.1371/journal.pone.0287226 (PMC10337867; doi:10.1371/journal.pone.0287226)
Supplement: S1 Table — Results describing associations with knowledge evolution, binarized as split or merge (1) and continuation or death (0), and all our exogenous variables. Note significant positive associations with language score and number of weak members, plus a negative association for the year. Mean network interdisciplinarity score (considering strong members only) is statistically insignificant, as is the number of weak members. Mean network interdisciplinarity score (considering weak members only) is insignificant, yet it has a significant marginal effect. Per common practice, we purposefully re-ran our analysis discarding insignificant variables, to evaluate significance of network score among weak members and confirm our findings on language score, number of weak members, and year. (PDF) [file pone.0287226.s002.pdf]

| | Model estimates | | | | | |
|---|---|---|---|---|---|---|
| **Model Input (per cluster)** | *Coeff.* | *Std. err.* | z | $P > \|z\|$ | *[0.025* | *0.975]* |
| Cluster size (considering strong members only) | 0.010 | 0.006 | 1.638 | 0.101 | -0.002 | 0.021 |
| Cluster size (considering weak members only) | 0.050 | 0.015 | 3.107 | 0.002 | 0.017 | 0.075 |
| Mean language ID score | 2.704 | 1.189 | 2.274 | 0.023 | 0.374 | 5.035 |
| Mean network ID score (considering strong members only) | 9.960 | 5.105 | 1.951 | 0.051 | -0.046 | 19.966 |
| Mean network ID score (considering weak members only) | -3.801 | 4.878 | -0.779 | 0.436 | -13.361 | 5.759 |
| Year | -0.179 | 0.072 | -2.495 | 0.013 | -0.320 | -0.038 |
| Constant | 358.426 | 144.313 | 2.484 | 0.013 | 75.578 | 641.274 |

| | Marginal effects | | | | | |
|---|---|---|---|---|---|---|
| **Model Input (per cluster)** | $dy/dx$ | *Std. err.* | $z$ | $P > \|z\|$ | *[0.025* | *0.975]* |
| Cluster size (considering strong members only) | 0.002 | 0.001 | 1.660 | 0.097 | -0.000 | 0.005 |
| Cluster size (considering weak members only) | 0.010 | 0.003 | 3.281 | 0.001 | 0.004 | 0.016 |
| Mean language ID score | 0.580 | 0.247 | 2.351 | 0.019 | 0.096 | 1.063 |
| Mean network ID score (considering strong members only) | 2.134 | 1.070 | 1.995 | 0.046 | 0.038 | 4.231 |
| Mean network ID score (considering weak members only) | -0.815 | 1.042 | -0.782 | 0.434 | -2.856 | 1.227 |
| Year | -0.039 | 0.015 | -2.589 | 0.010 | -0.067 | -0.009 |
