# Supplementary material for: The evolution of scientific literature as metastable knowledge states
Source: PLoS One. 2023 Jul 12;18(7):e0287226. doi: 10.1371/journal.pone.0287226 (PMC10337867; doi:10.1371/journal.pone.0287226)
Supplement: S2 Table — (PDF) [file pone.0287226.s003.pdf]

Discipline	# clusters	# publications	Task 1: Mean Jaccard similarity	Task 2: Evaluation of algorithm-generated clusters (scale of 1-5)
Marketing	4	13	0.46	4
Psychology	3	12	0.7	4
Political Science	3	12	0.88	5
