# Supplementary figures and images for: The evolution of scientific literature as metastable knowledge states
Source: PLoS One. 2023 Jul 12;18(7):e0287226. doi: 10.1371/journal.pone.0287226 (PMC10337867; doi:10.1371/journal.pone.0287226)

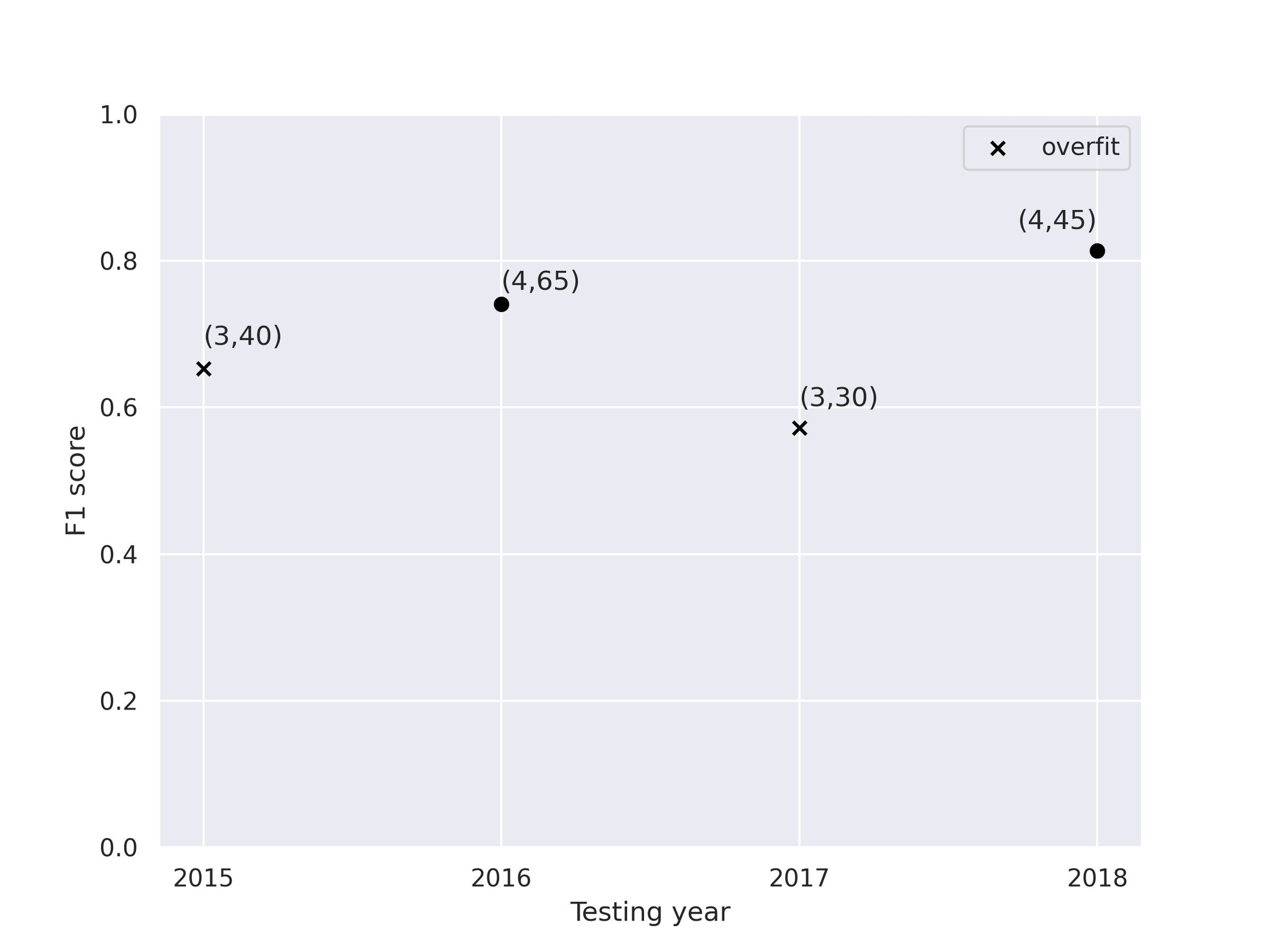

Supplement: S1 Fig — Plot showing the best F1 scores achieved on the test data when random forest model trained by varying the sizes of training data on the events occurring in the following year. The labels shown next to each data point represent the model parameters (maximum depth, number of classifiers). When trained on events occurring between 2011 and 2014, every model in the grid search overfit to the training data. (TIF) [file pone.0287226.s004.tif]

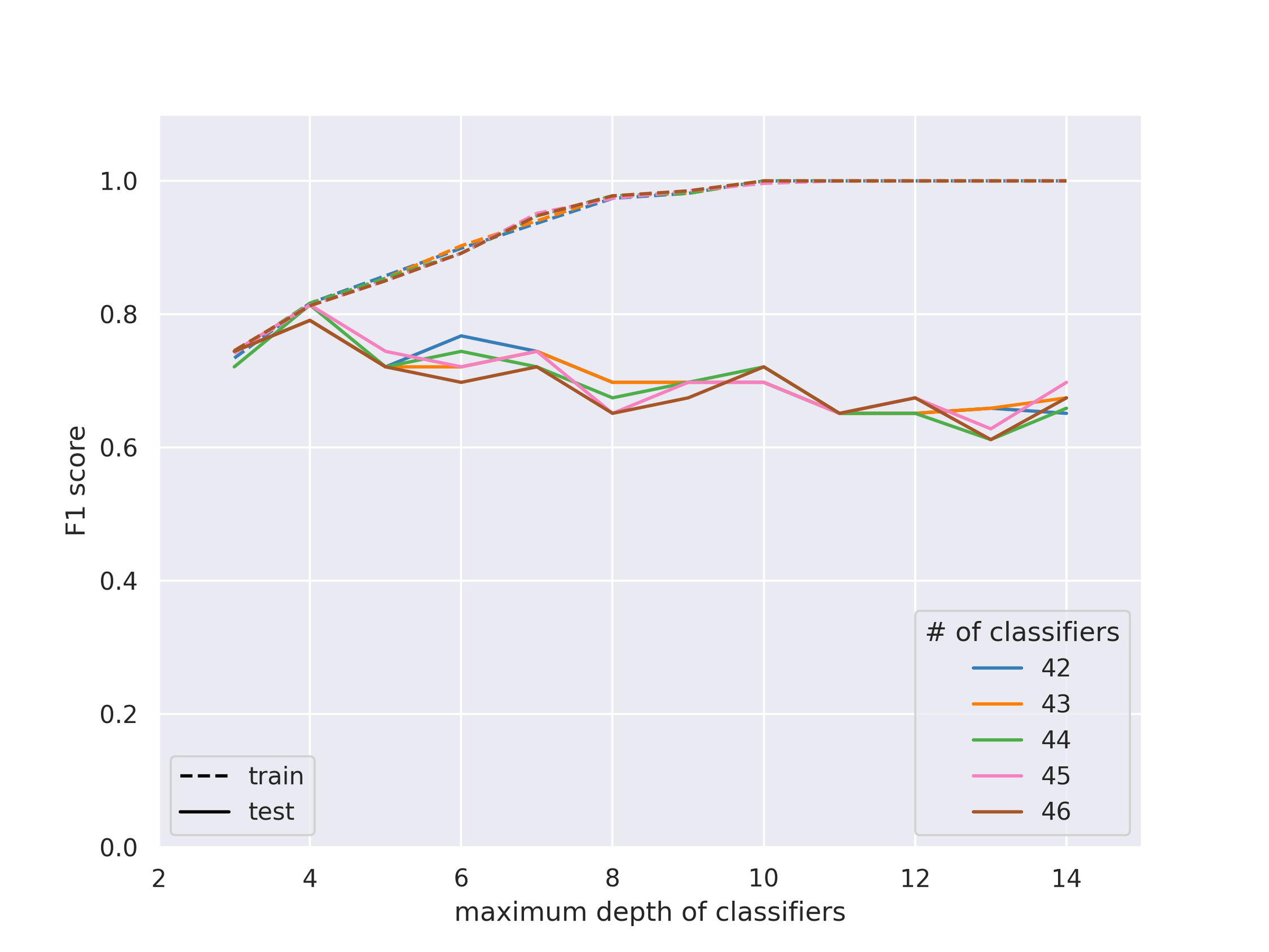

Supplement: S2 Fig — Line plot showing the random forest model F1 scores achieved when events in 2011-17 were used for training and tested on predicting 2018 events for a sample of model parameters (maximum depth, number of classifiers) from grid search. The plot shows that the maximum depth of classifier trees has a higher effect on generalizability of the model than number of classifiers. The model performance on held out data diverges from the performance on training data due to the overfitting as maximum depth increases. (TIF) [file pone.0287226.s005.tif]

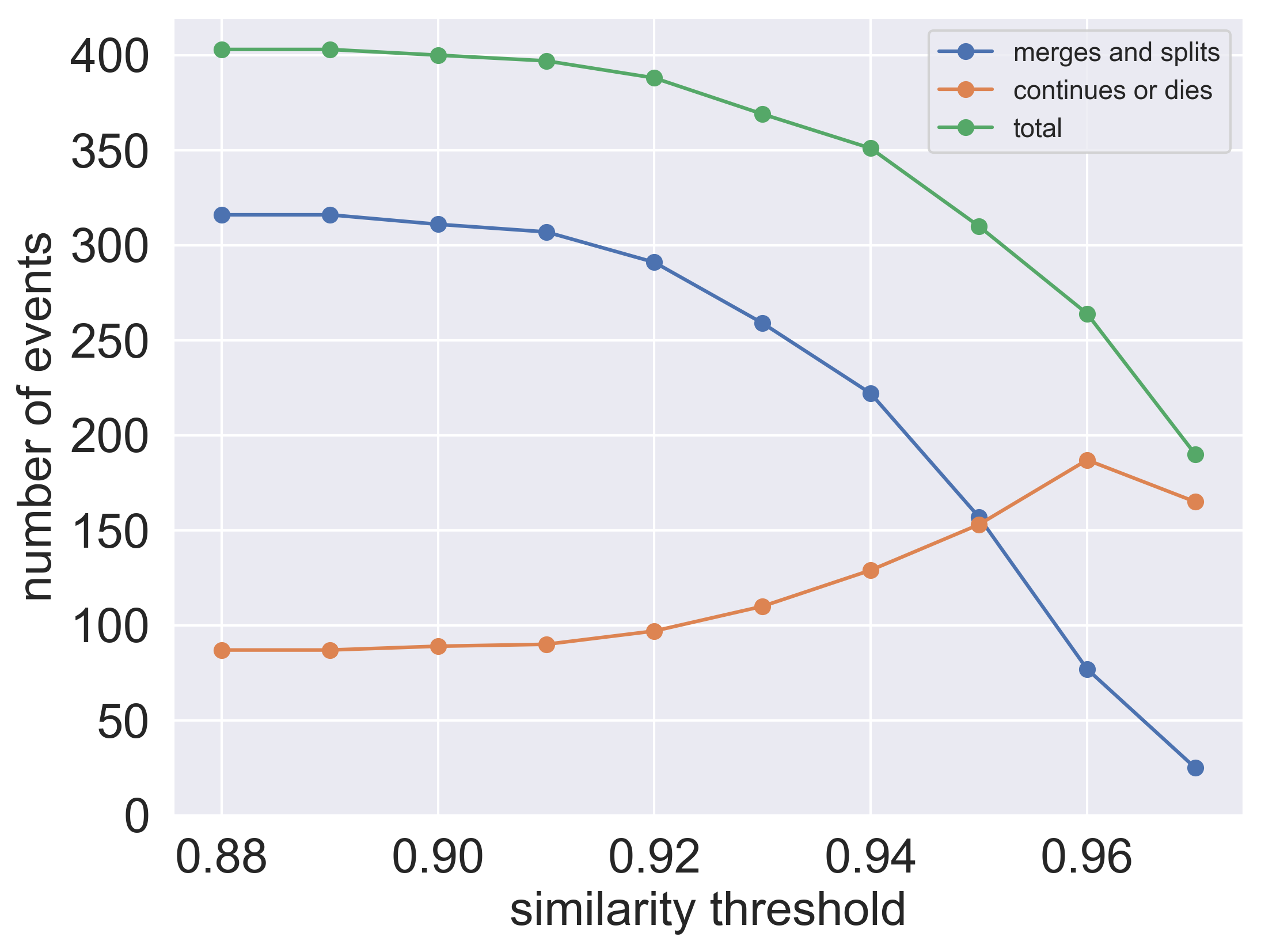

Supplement: S3 Fig — (TIF) [file pone.0287226.s006.tif]

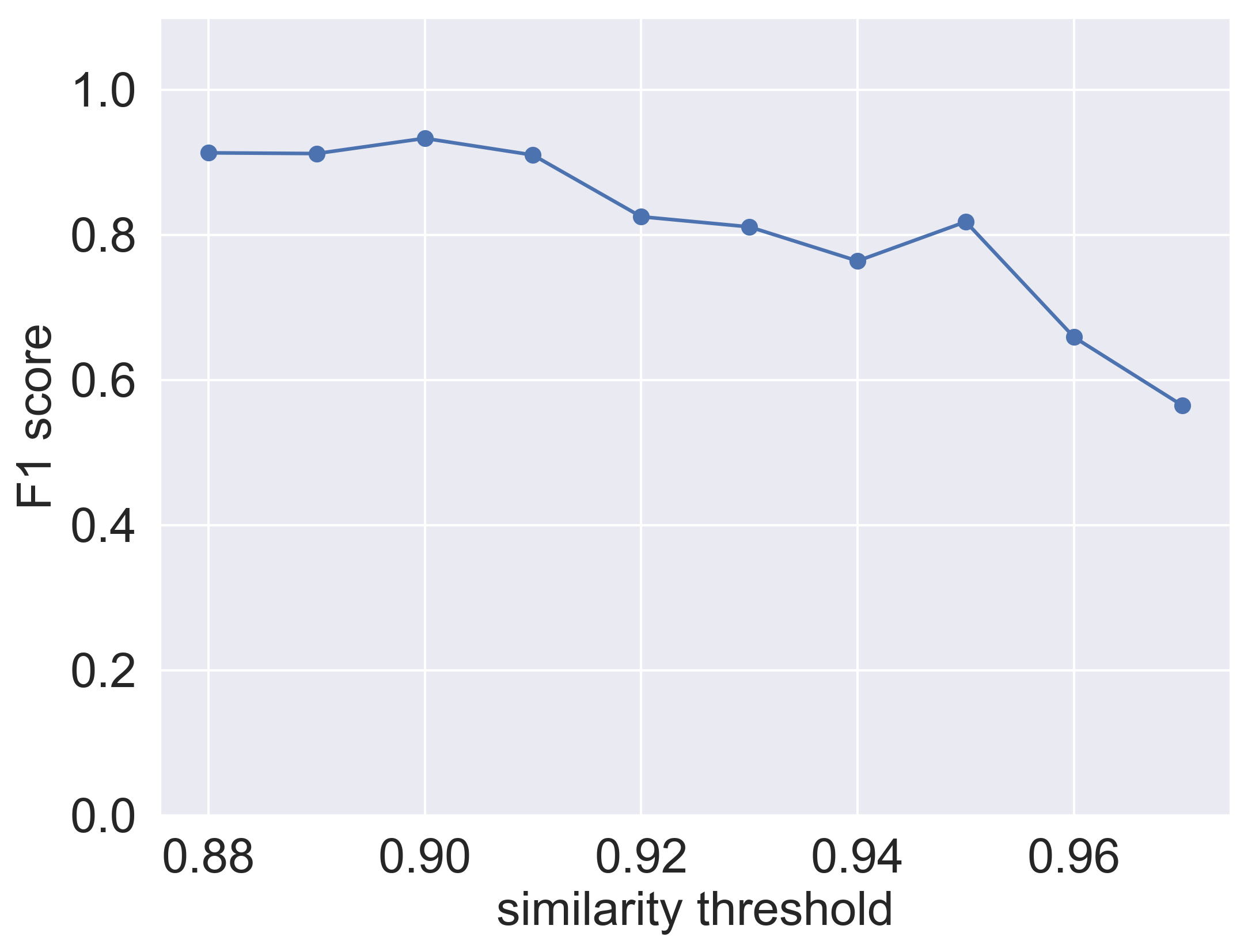

Supplement: S4 Fig — (TIF) [file pone.0287226.s007.tif]
