# Supplementary material for: The evolution of scientific literature as metastable knowledge states
Source: PLoS One. 2023 Jul 12;18(7):e0287226. doi: 10.1371/journal.pone.0287226 (PMC10337867; doi:10.1371/journal.pone.0287226)
Supplement: Attachment — Submitted filename: Response to reviewers.pdf [file pone.0287226.s008.pdf]

Response to Reviews

We thank the editors and the reviewers for their consideration and their thoughtful and helpful comments. We have implemented a number of revisions to the work and the paper based on your suggestions, and the paper is stronger now because of it. Following, please find point-by-point response to each of the editor's and reviewers' comments. We have colored our responses in blue for ease of reading, and likewise corresponding changes to the manuscript are also provided in blue.

Response to Editor's comments

1. Please ensure that your manuscript meets PLOS ONE's style requirements, including those for file naming.

   Thank you. The manuscript now meets the style requirements, per the link you have provided.

2. Thank you for stating the following financial disclosure: This research was supported by the National Center for Science and Engineering Statistics (NCSES) at the National Science Foundation through award 49100420C0030. The study funders consulted with the authors throughout the project timeline.

   Please state what role the funders took in the study. If the funders had no role, please state: "The funders had no role in study design, data collection and analysis, decision to publish, or preparation of the manuscript." If this statement is not correct you must amend it as needed. Please include this amended Role of Funder statement in your cover letter; we will change the online submission form on your behalf.

   The funders had no role in study design, data collection and analysis, decision to publish, or preparation of the manuscript. We have provided the amended Financial Disclosure Statement in our cover letter, per request.

3. Thank you for stating the following in the Acknowledgments Section of your manuscript: This research was supported by the National Center for Science and Engineering Statistics (NCSES) at the National Science Foundation through award 49100420C0030. However, funding information should not appear in the Acknowledgments section or other areas of your manuscript. We will only publish funding information present in the Funding Statement section of the online submission form. Please remove any funding-related text from the manuscript and let us know how you would like to update your Funding Statement. Currently, your Funding Statement reads as follows: This research was supported by the National Center for Science and Engineering Statistics (NCSES) at the National Science Foundation through award 49100420C0030. The study funders consulted with the authors throughout the project timeline. Please include your amended statements within your cover letter; we will change the online submission form on your behalf.

   We have removed funding information from the Acknowledgements section of the manuscript. We have provided the amended Financial Disclosure Statement in our cover letter, per request.

4. We note that you have indicated that data from this study are available upon request. PLOS only allows data to be available upon request if there are legal or ethical restrictions on sharing data publicly. For more information on unacceptable data access restrictions, please see In your revised cover letter, please address the following prompts: a) If there are ethical or legal restrictions on sharing a de-identified data set, please explain them in detail (e.g., data contain potentially sensitive information, data are owned by a third-party organization, etc.) and who has imposed them (e.g., an ethics committee). Please also provide contact information for a data access committee, ethics committee, or other institutional body to which data requests may be sent. b) If there are no restrictions, please upload the minimal anonymized data set necessary to replicate your study findings as either Supporting Information files or to a stable, public repository and provide us with the relevant URLs, DOIs, or accession numbers. For a list of acceptable repositories, please see We will update your Data Availability statement on your behalf to reflect the information you provide.

   The publication DOIs used as the seeds to collect data represent a sufficient dataset to replicate our findings, either directly using Web of Science (for anyone with a license) or indirectly using Semantic Scholar (which is open source). We have added the link to the Data Availability statement in the manuscript.

5. Please review your reference list to ensure that it is complete and correct. If you have cited papers that have been retracted, please include the rationale for doing so in the manuscript text, or remove these references and replace them with relevant current references. Any changes to the reference list should be mentioned in the rebuttal letter that accompanies your revised manuscript. If you need to cite a retracted article, indicate the article's retracted status in the References list and also include a citation and full reference for the retraction notice.

   Thank you. We have carefully reviewed the reference list. It is complete and correct.

Response to Reviewers' Evaluations

1. Is the manuscript technically sound, and do the data support the conclusions?

   The manuscript must describe a technically sound piece of scientific research with data that supports the conclusions. Experiments must have been conducted rigorously, with appropriate controls, replication, and sample sizes. The conclusions must be drawn appropriately based on the data presented.
   Reviewer #1: Partly
   Reviewer #2: Yes

   To further ensure the technical soundness of the work, we have added ablation studies, modified the sizes of train and test datasets, and used grid search to avoid overfitting of the classification model. These changes were based on reviewers' suggestions. Further detail on these changes is provided below, in **Response to Reviewers' Comments to the Authors**.

2. Has the statistical analysis been performed appropriately and rigorously?
   Reviewer #1: Yes
   Reviewer #2: Yes

3. Have the authors made all data underlying the findings in their manuscript fully available?

   The PLOS Data policy requires authors to make all data underlying the findings described in their manuscript fully available without restriction, with rare exception (please refer to the Data Availability Statement in the manuscript PDF file). The data should be provided as part of the manuscript or its supporting information, or deposited to a public repository. For example, in addition to summary statistics, the data points behind means, medians and variance measures should be available. If there are restrictions on publicly sharing data—e.g. participant privacy or use of data from a third party—those must be specified.
   Reviewer #1: No
   Reviewer #2: Yes

   We have now added the data file consisting of seed DOIs that are used for collecting the data from the scientific data sources (Web of Science/Semantic Scholar). We have added the file to a public github repo and added the link to the Data Availability Statement.

4. Is the manuscript presented in an intelligible fashion and written in standard English?

   PLOS ONE does not copyedit accepted manuscripts, so the language in submitted articles must be clear, correct, and unambiguous. Any typographical or grammatical errors should be corrected at revision, so please note any specific errors here.
   Reviewer #1: No
   Reviewer #2: Yes

   We have done a thorough proofread and copy-edit of the manuscript.

Response to Reviewers' Comments to the Authors

Reviewer #1:

To my understanding, this is a study paper. Some of the methods presented in this paper can be questionable but the study and direction of the study is interesting.

Thank you for the comments. We have made some changes to the methods - specifically, we have:

1) added ablation studies – manuscript Section **S1 Appendix**, and referenced in Section **Results**;

2) demonstrated robustness of the methods with respect to varied selection of train and test datasets – manuscript Section **S1 Appendix**, and referenced in Section **Materials and Methods**;

3) and, used grid search to avoid overfitting of the classification model – manuscript Section **S1 Appendix**, and referenced in Section **Materials and Methods**.

There are several missing citations and grammatical errors. Hence I do advise the authors to revise the paper and correct the mistakes.
We have done a thorough proofread and copy-edit of the manuscript.

The paper itself is not so strong, however the study might get researchers to think about some of the mentioned methodology which can lead to future discoveries.
We do believe that the methodological changes and additions we have made have made the paper stronger. We also appreciate and agree with your comment on methodology. Given the bibliometrics literature's longstanding focus on citation networks, we highlight the importance of novel methods for modeling text and networks together for understanding scientific trends and dynamics.

Based on the taxonomy, if a death occurs at time t but reappears at time t+1, how would you explain it?
Thank you for the interesting question. You are right – it is possible that a knowledge cluster (topic/discipline) could reappear at a future timestep after it has "died". We did not investigate this possibility when designing the current study. However, the results from the study allowed us to post-hoc manually check for the reappearance of dead clusters. To do so, we calculated pairwise cosine similarities between the representative language embeddings of all dead clusters to all clusters appearing in the later time steps. Based on the definition of the evolutionary events, we have used the similarity threshold of 0.95 to consider a dead cluster as having "reappeared". We did not observe any of these cases in our dataset when we did this check. However, this is a possibility that should be noted, and we have now included reference to it as a footnote in Section **Materials and methods**.

Reviewer #2:

The manuscript "The evolution of scientific literature as metastable knowledge states" proposes a novel method of combining both citation- and language-based techniques to follow the changes in scientific literature. Authors use a combination of time-tested and modern methods to perform feature engineering and clustering and formulate a machine learning problem of predicting the state of a cluster in the future. They show that both language and network-derived features have significant importance in this prediction process.
Pros:
- Rigorous statistical analysis of the results
- Good pipeline summary and overall narrative
- Clear motivation
- Popular (and well-documented) frameworks, ideas and techniques used

Details I would pay additional attention to:
- Figure 3 is quite hard to read. Not clear what layout was used.

We believe the difficulty may have been related to all of the grey nodes in the previous image. We have revised the image suppressing the grey nodes and we have made note of this in Section **Materials and methods**.

When the authors describe their ML problem, they split the data into train/test set a single time: events from 2011-2017 are used for training and from 2018 for test. It could be that this particular split is not stable and may show different results if another option is taken (say, 2011-2015 for training and 2016 for test or something similar).
We appreciate the concern. We have now conducted additional experiments to address this issue. The results of which are presented in Appendix. While training with events from 2011 to 2017 does offer best performance, training on events 2011 to 2015 also offer good performance. However, the other splits (2011 - 2014 and 2011 - 2016) lead to overfitting of models.

Authors say that they used a scikit-learn Random Forest implementation with default hyperparameter values.

From my experience, the provided default values tend to make RF overfit and require optimization.
Thank you very much for this helpful suggestion. We have now implemented grid search to avoid overfitting and found the optimal parameters for the model. The observations from the parameter search experiments were reported in the Appendix section of the article. Using the optimal parameters has improved the prediction metrics. Thank you once again.

The dataset description part does not include any information about how many samples were taken from each of the "prominent journals" and how this number evolves over time. It may affect clustering results, especially in a dynamic scenario.
We apologize for the lack of detail here. We borrow from the sample collected in conjunction with the DARPA SCORE program. The sample contains approximately equal representation of papers per year and per journal. The complete list of journals is provided in Supplement Table 1 here: https://osf.io/preprints/socarxiv/46mnb/. This is now stated and referenced within the **Dataset** section of our paper.

Authors mention that they used "multi-objective Bayesian hyperparameter tuning for the UMAP-HDBSCAN pipeline" but they did not include the optimization target, so it's not quite clear what would be considered optimal. It significantly affects the clustering itself as well as its input data (from UMAP dimensionality reduction framework).
We now provide additional details related to the hyperparameter tuning process in the **SPECTER-based topic modeling** subsection.

I would like to see the number of clusters over time. The authors only provided the total number of clusters (371).
The distribution of number of language-based clusters as well as network communities is shown in the inset plot in Figure 5 of the manuscript.

I would also like to see ablation studies (what if only network-based or language based ID scores are used?) and corresponding classification scores.
Thank you for the suggestion. We have now conducted ablation studies and reported the results in the **S1 Appendix** of the manuscript, and referenced them in Section **Results** of the main text. The results of the ablation studies show that performance of the models trained using either language- or network-based features is lower than combined performance, but also offers insights into the power of SPECTER-based language modelling.

Verdict: It is a well-written paper with promising ideas, reasonably easy-to-follow technical part and good illustrations (for the most part). However, I believe that it is possible to get more fruitful results from the obtained preprocessed data and calculated features.
We appreciate this comment and we agree that the additions you have suggested (train/test splits, ablation studies, hyperparameter tuning) have made the paper stronger now.

I am also curious to see how this approach performs on a larger scale dataset and how the topic granularity is taken into account.
This is an interesting question. We agree that this approach should continue to be tested on other datasets and refined in future work. With respect to *which* datasets should be next, we have a few thoughts:
Generality: This dataset is relatively large compared to many in the bibliometrics literature. It is, therefore, also quite general to the social and behavioral sciences broadly. We would suggest that the most important next step would be to test this approach on a smaller dataset but one where some better measures of ground truth are available. In the early days of developing these methods, we tested on much smaller samples in domains where we (authors) personally had expertise, e.g., network science, machine learning. We did this so we could have a sanity check when we looked at our clusters and interdisciplinarity metrics anecdotally. We imagine that "one-size-fits-all" models are helpful from an intuition perspective, but are going to necessarily lack specificity.
Computational complexity: This approach should scale to significantly larger corpuses and we have indeed run the pipeline on the full Web of Science corpus starting in 2005, but at that scale (millions of papers per year) the clustering algorithm requires careful selection of hyperparameter distributions to avoid extremely long per-trial convergence times.
We have added an abbreviated version of these thoughts to the paper's **Discussion** section.

Some technical details were (deliberately?) omitted by authors, but I believe they can include them if they find it necessary.

Thank you for this suggestion. We have now added technical details about clustering and hyperparameter tuning in Section **Materials and Methods**. We believe that the current level of detail is sufficient to allow other researchers to replicate our approach.
