# Supplementary material for: The evolution of scientific literature as metastable knowledge states
Source: PLoS One. 2023 Jul 12;18(7):e0287226. doi: 10.1371/journal.pone.0287226 (PMC10337867; doi:10.1371/journal.pone.0287226)
Supplement: Attachment — Submitted filename: ResponseToReviews.pdf [file pone.0287226.s009.pdf]

Response to Reviews

We thank the reviewers for their thoughtful and helpful comments. We have implemented a number of revisions to the work and the paper based on these suggestions, and the paper is stronger now because of it. Following, please find point-by-point responses to each of the reviewers' comments. Our responses are denoted with blue text; likewise, changes to the manuscript are also provided in blue.

Reviewers' comments

Reviewer's Responses to Questions Comments to the Author

1. If the authors have adequately addressed your comments raised in a previous round of review and you feel that this manuscript is now acceptable for publication, you may indicate that here to bypass the "Comments to the Author" section, enter your conflict of interest statement in the "Confidential to Editor" section, and submit your "Accept" recommendation.
Reviewer #2: All comments have been addressed
Reviewer #3: (No Response)
Reviewer #4: (No Response)

2. Is the manuscript technically sound, and do the data support the conclusions?
The manuscript must describe a technically sound piece of scientific research with data that supports the conclusions. Experiments must have been conducted rigorously, with appropriate controls, replication, and sample sizes. The conclusions must be drawn appropriately based on the data presented.
Reviewer #2: Yes
Reviewer #3: Yes
Reviewer #4: Yes

3. Has the statistical analysis been performed appropriately and rigorously?
Reviewer #2: Yes
Reviewer #3: Yes
Reviewer #4: Yes

4. Have the authors made all data underlying the findings in their manuscript fully available?
The PLOS Data policy requires authors to make all data underlying the findings described in their manuscript fully available without restriction, with rare exception (please refer to the Data Availability Statement in the manuscript PDF file). The data should be provided as part of the manuscript or its supporting information, or deposited to a public repository. For example, in addition to summary statistics, the data points behind means, medians and variance measures should be available. If there are restrictions on publicly sharing data—e.g. participant privacy or use of data from a third party—those must be specified.
Reviewer #2: Yes
Reviewer #3: No
Reviewer #4: No
We have provided the DOIs and titles corresponding to all seed papers analysed in the study at `https://github.com/QS-2/VESPID/blob/main/seed_data/score_papers.csv`. While we are not able to directly share the raw data (titles and abstracts) used for our analysis due to our licensing agreement with Web of Science, this list of DOIs is sufficient to replicate our findings, either directly using Web of Science (for anyone with a license) or indirectly using Semantic Scholar, which is open source. This is described in the Data Sharing section of the manuscript.

5. Is the manuscript presented in an intelligible fashion and written in standard English?
PLOS ONE does not copyedit accepted manuscripts, so the language in submitted articles must be clear, correct, and unambiguous. Any typographical or grammatical errors should be corrected at revision, so please note any specific errors here.

Reviewer #2: Yes
Reviewer #3: Yes
Reviewer #4: Yes

6. Review Comments to the Author
Please use the space provided to explain your answers to the questions above. You may also include additional comments for the author, including concerns about dual publication, research ethics, or publication ethics. (Please upload your review as an attachment if it exceeds 20,000 characters)

Reviewer #2: This is my short review of the first revision of the manuscript "The evolution of scientific literature as metastable knowledge states". In my opinion, the authors addressed the comments I left in my first review. From the technical standpoint, paper looks more explicit and transparent now.

However, I still have some additions considering the manuscript:

1. When the citation network is constructed, the authors use a larger dataset of papers because they aim to capture the information about references. But they only use the reference section of the papers (from what I understand from this sentence: "For citation network analyses, we also included all papers referenced by these papers."). My concern is that this approach may not be comprehensive because it does not contain the information about the incoming connections (WHO cites a given paper, e.g. "Cited by" button in Google Scholar). I understand that this data may be harder to obtain and process, but that this improvement could be beneficial for citation network analysis.
Thank you for raising this concern. Our reasoning behind not including the incoming citations to a publication is because we are using historical information to forecast the state of a cluster/discipline in the following year. Since it takes time for citations to accumulate, we assume that publications' incoming citations wouldn't be known at the time of forecasting.

2. I would like to get a better understanding of "weak members" of the clusters. Since the authors mention that "HDBSCAN refers to these non-confident assignments as noise", which is true, they also have expectations about novelty these outliers can bring: "We expect these not to be noise in the traditional sense (e.g., an outlier or data worthy of discarding as it provides no analytical value) but instead to potentially add value as novel research." I would like the authors to emphasize on this claim and maybe provide some examples of their point of view and why they think it is valid. I personally would like to see examples, where these "weak members" at timestamp t form a stable cluster at timestamp t+1. It is also interesting to investigate their stability: how they behave over time and over different clustering (UMAP + HDBSCAN) runs. These papers, being outliers, can simply be a result of some edge cases for pre-trained embeddings. For example, some research areas could be covered worse than others resulting in underrepresented "spots" in the latent space.
Thank you for this insightful comment.

- Our assumptions about novelty stem from the observation that papers identified as weak members are *different* from the ones that were confidently assigned to a cluster. By novelty we meant that the content of these weak members is different from other work in their field. For example, one weak member is an article titled *Must psychologists change the way they analyze their data?* (DOI: 10.1037a0024777) published in *Journal of Personality and Social Psychology.* Another is titled *Strengthening the Experimenter's Toolbox: Statistical Estimation of Internal Validity* (10.1111j.15405907.2011.00576.x) published in *American Journal of Political Science.* These papers are devoted to statistical methods and are published in venues that predominantly publish practical applications. However, we agree that a claim about novelty requires substantiation beyond anecdotal evidence so we have now modified this discussion in the manuscript.
- We expect that weak members at time t_1 can lead to a stable cluster at t_2 ($t_2 > t_1$) only if the ensuing research efforts result in a "critical mass". We plan to explore this in future work and have included this point within the manuscript's discussion section.
- The UMAP + HDBSCAN clustering pipeline is a standard approach in the literature for clustering text, but the question of its stability is important. To address this question, we have done some testing on a subset of the data to assess the stability of our results. Specifically, we ran the UMAP algorithm with

five different random seeds on our 2011 data. To compare results between runs, we calculated the jaccard similarity index. We find a mean similarity of 0.78 (pairwise, between runs) with standard deviation 0.042. Results are fully repeatable (jaccard index of 1) when the random seed is kept constant across the runs.

- We agree that the dataset used for pre-training the SPECTER model affects the resulting embeddings. We have now added text acknowledging this as a limitation for this work.

3. One of my previous comments was about large scale experimentation and scaling. By saying that, I mean the size of textual embedding space as well. At this moment, textual embeddings (then dimensionality reduction and clustering) are applied to only less than 20,000 papers, which I cannot refer to as a "large scale" experiment (I am expecting at least a million papers there). The citation network scales differently, but it is still a relatively small (and very sparse) graph. It would be interesting if the authors could replicate their results using some faster clustering algorithm (i.e., k-means) and experiment with larger datasets in their future work.
Thank you for these comments.

- We agree that the scale of the current dataset is relatively small compared to general machine learning benchmarks in other domains. In ongoing work, we are streamlining the computational pipeline as well as collecting more data with an aim to similarly investigate the full Web of Science corpus.

- We also agree that future work should compare clusters output via our approach to other benchmark clustering algorithms. To our knowledge, there would not be a clear "best" candidate. In addition to requiring an a prior input value for k, k-means clustering assumes the clusters to be spherical which may not be appropriate in the case of high-dimensional knowledge clusters. However, these questions are important to study more formally and we have included a note on this in our discussion section.

Verdict: I think that he authors made some improvements with their first revision, but there are still several things to address before the manuscript can be fully accepted. If they believe that more experimentation is not feasible/realistic/necessary to perform, I would gladly ask them to add some comments on why the particular decisions were made.

Reviewer #3: This paper uses a large data set of papers and citations to examine how scientific ideas evolve over time. The paper was well explained with interesting figures. The overall results made sense and were interesting.

There was no mention of the common errors in citations in the limitations. Likely this measurement error would not be a big issue given the sample size, but it should be mentioned. See for example, DOI: 10.1042/CS20201573.
We appreciate the comment we have now added text related to the errors in citation analysis.

The reduction in clusters over time (figure 5) could be because of movements outside the selected fields that are not captured. For example, for the not included field "Computer Science" there could have been a cluster building in the early years that was then joined by researchers from one of the selected fields. I don't believe that this kind of movement would be captured by this study design. It is counter-intuitive that ideas have decreased over time, and no explanation is given.
We thank the reviewer for raising this idea.

- Yes, we have constrained our analyses to the social sciences and we agree that movements from outside this set of fields could be causing shifts within our data that we may not be able to observe, e.g., increasing integration between computer science and social sciences. We have added this to our discussion of limitations.

- We think that reduction of ideas, at the final years of dataset, may be a misunderstanding. The number of language clusters, which represent ideas, hovers roughly around 50, except for 2015 (see Figure 5). However, the number of network communities do decrease for the last two years in the dataset, which we believe is what the reviewer is referring to. We presented the number of network communities to demonstrate that there is some but not a complete overlap between the network communities and the language clusters. However, the network communities are not otherwise used in our analyses.

There are a lot of analytical choices in the paper, hence there is likely to be some model uncertainty (DOI: 10.1111/j.1467-9574.2012.00530.x). Other researchers may have made different and equally plausible decisions that could give different results. I think this should be acknowledged as a limitation.
We have now added the limitations to the discussion section.

This is not multinomial logistic regression (line 248) as that is for outcomes with multiple classes. The outcome here is binary, so this is simply logistic regression.
Thank you for pointing this out. We have updated the manuscript.

The authors are not compliant with the PLOS ONE data sharing policy: "The PLOS Data policy requires authors to make all data underlying the findings described in their manuscript fully available without restriction, with rare exception"
We have provided the DOIs and titles corresponding to all seed papers analysed in the study at `https://github.com/QS-2/VESPID/blob/main/seed_data/score_papers.csv`. While we are not able to directly share the raw data (titles and abstracts) used for our analysis due to our licensing agreement with Web of Science, this list of DOIs is sufficient to replicate our findings, either directly using Web of Science (for anyone with a license) or indirectly using Semantic Scholar, which is open source. This is described in the Data Sharing section of the manuscript.

Minor comments

- Line 36 "state-of-the-art" is hyperbole. Point out the benefits of the methods.
  We have now modified the language around the claim.
- Line 80, why were these fields chosen?
  We have chosen these disciplines because the dataset was carefully curated through stratified sampling for a different project, Systematizing Confidence in Open Research and Evidence (DARPA SCORE), that aimed to ensure an accurate representation of the disciplines within the social and behavioral sciences.
- What is embedding? Line 98.
  We have now modified the language.
- SPECTER may "outperform" other methods, but how accurate is it? Line 101
  Our assertion relating outperforming comes from the findings provided in the SPECTER article (DOI 10.48550arXiv.2004.07180). However, we acknowledge the need for clearer language and have consequently revised the statement.
- There are a lot of acronyms in the paper. I am fairly certain that DBCV is not defined.
  We apologize for the lack of definition. We have now added it to the manuscript.
- The overall proportion of weak members would be interesting (figure 2 legend)
  We have now added this information to the legend (approx 11.06%).
- Figure 2 was interesting, but I didn't know what the x- and y-axes were.
  We have now added the text in legend. Since the x- and y-axes for this figure were created by projecting the semantic embeddings to two dimensions using UMAP, the axes are no longer interpretable.
- Could some joins between fields be due to commonly used methods papers?
  That is an interesting idea. We agree that the merge event could happen due to shared methods. We hypothesize that the commonality between the fields should reach a certain "critical mass" for merging to occur and plan to analyze this in a future study.
- I found it surprising that the death of an area was labelled as "stable", as that seems like a definite change. This is just a wording issue.
- Figure 4. The paper for the top-right circle is missing a title.
  Thank you for pointing this we have now added a title for the publication node in the schematic diagram.

- The splits are based on annual data. That is probably suitable, but should be mentioned as a limitation. We agree that the choice of one year as a time step fails to capture the evolutionary dynamics with cycles less than a year. We have now added this as a limitation.

- "features were not significant" (table 1 legend) best to focus on the size of the difference rather than the statistical significance (DOI 10.1080/00031305.2016.1154108)
  Thank you for this point and the reference. We assume by "focusing on the size of the difference" you mean the difference in the effect sizes. If so, we would need to threshold values for selection based on the marginal effects while considering the standard errors simultaneously. We have added this in the manuscript as a limitation.

- "including only features shown to be statistically significant" there are better model selection methods available than using statistical significance, see for example Statistical Learning with Sparsity: the Lasso and Generalizations by Trevor Hastie, Robert Tibshirani and Martin Wainwright (May 2015)
  Our approach to feature selection is commonly used for our choice of the model - specifically, logistic regression (ISBN:9780470582473). We agree and have noted in the manuscript that future work should address the important question of choosing the best statistical model to capture these phenomena.

Reviewer #4: The authors present an integrative approach to combine semantic embedding models and network models of scientific literature. The evolution of scientific literature can then be modeled in terms of a stream of dynamic events over time.

The authors made some good use of some of the recent advances in relevant fields and constructed a meaningful pipeline.

In general, the paper is interesting to read and various technical decisions are explained relatively well.

There are some areas I'd like to draw authors' attention for possible clarification and further improvements.

Relevant works cited by the authors are okay, but there are more closely related works that authors may not be aware of. They should be incorporated to strengthen the work. For example, there is a substantial body of the literature that underlines the immediate context of the work, including graph embedding, theories and mechanisms of how science advances, characterizing clusters with keyphrases, and time slicing the literature to trace the evolution.
We have now added literature as suggested.

The role of the concept of metastability in this paper is not adequately developed. In fact, it is so weak that it can be dropped altogether without losing anything.
We appreciate this point. We use the term metastability to offer a simple intuition for motivating ideas of this work. That is, the emergence of interdisciplinary work signals that the landscape is shifting within and around a field of study. To our knowledge, there is no better name for this idea. However, if the reviewer would prefer we can remove it.

There are a few other places where clarifications and additional explanations would be helpful. For example, the switch to interdisciplinarity in line 47 on page 2 is rather unanticipated. Existing ways to measure interdisciplinarity should be better documented and evaluated. For example, gini and Shannon entropy were mentioned but it is not clear why betweenness centrality is chosen.
That you for these comments, we have modified the manscript accordingly. Specifically:

- We have modified the text there (line 47) to better transition to interdisciplinarity.

- While we referenced the Shannon entropy and Gini index, we have developed our own measure of interdisciplinarity based on the semantic information. We took this approach since the interdiciplinary metrics based on Gini index as well as Shannon entropy use predermined subject categories (e.g. Web of Science categories) of the references of the publication rather than their semantic information. Moreover, for the network based interdisciplinarity metric we used the betweenness centrality because it relies on citation networks which cannot be captured by other metrics such as Gini index.

Page 3, the beginning of the paragraph mentioned that the dataset contains 19,177 papers published, but at the end of the paragraph, the complete dataset includes 839,096 papers, which can be confusing. How many

papers in the dataset and how many citations? Do you mean the 839,096 are references cited by the 19,177 papers in a total of 1.45 million instances? If this is indeed the case, it would be helpful to differentiate the 19,177 papers vs 839,096 papers by calling the latter as references.
Thank you for the comment. We agree that the prior description was unclear. Our study analyzes our core dataset, containing 19,177 publications. We have added the references cited by the 19,177 publications in our dataset to study the citation context for network analysis. We have now added a clear explanation to the manuscript.

DBCV needs to be defined.
Thank you. We apologize for neglecting to include this definition. We have now added it to the manuscript.

The way you are talking about interdisciplinarity can be misleading. It seems more similar to something like inter-cluster connectivity, i.e. more precisely these are cluster-based, whereas it would be questionable to claim these clusters are discipline equivalent. I'd recommend you consider a more precise name for the metric in this context.
Thank you for the thoughtful point. We acknowledge that the clusters may not represent disciplines. There is no consensus in the research community about the definition and operationalization of interdisciplinarity (see, e.g., `https://doi.org/10.1162/qss_a_00011`). Nevertheless, it is widely appreciated that interdisciplinarity is concerned with the integration of knowledge. Thus, we expect that our proposed metric can serve as an effective means of measuring interdisciplinarity, without relying on preconceived disciplinary boundaries. Usage of pre-defined subject categories can be a limitation particularly in light of studies indicating that scientific research is becoming more interdisciplinary (`https://doi.org/10.1007/s11192-008-2197-2`). For instance, a research article about usage of machine learning methods in bridge design can be classified as belonging to the pre-defined category of *civil engineering* if published in a journal specific to civil engineering. These subtle distinctions, which are frequently overlooked in the context of conventional disciplinary categories, can be captured by our proposed metric. To provide this context to the readers, we have updated the manuscript.

The use of the text-based dynamic event taxonomy is interesting.

7. PLOS authors have the option to publish the peer review history of their article (what does this mean?). If published, this will include your full peer review and any attached files.

If you choose "no", your identity will remain anonymous but your review may still be made public.

Do you want your identity to be public for this peer review? For information about this choice, including consent withdrawal, please see our Privacy Policy.
Reviewer #2: No
Reviewer #3: Yes: Adrian Barnett
Reviewer #4: No

[NOTE: If reviewer comments were submitted as an attachment file, they will be attached to this email and accessible via the submission site. Please log into your account, locate the manuscript record, and check for the action link "View Attachments". If this link does not appear, there are no attachment files.]

While revising your submission, please upload your figure files to the Preflight Analysis and Conversion Engine (PACE) digital diagnostic tool, https://pacev2.apexcovantage.com/. PACE helps ensure that figures meet PLOS requirements. To use PACE, you must first register as a user. Registration is free. Then, login and navigate to the UPLOAD tab, where you will find detailed instructions on how to use the tool. If you encounter any issues or have any questions when using PACE, please email PLOS at figures@plos.org. Please note that Supporting Information files do not need this step.
