# Supplementary material for: The evolution of scientific literature as metastable knowledge states
Source: PLoS One. 2023 Jul 12;18(7):e0287226. doi: 10.1371/journal.pone.0287226 (PMC10337867; doi:10.1371/journal.pone.0287226)
Supplement: Attachment — Submitted filename: ResponseToReviews3.pdf [file pone.0287226.s010.pdf]

Response to Reviews

We thank the reviewers for their thoughtful and helpful comments. We have implemented a number of revisions to the work and the paper based on these suggestions. Following, please find point-by-point responses to each of the reviewers' comments. Our responses are denoted with blue text; likewise, changes to the manuscript are also provided in blue.

Reviewers' comments

Reviewer's Responses to Questions
Comments to the Author

1. If the authors have adequately addressed your comments raised in a previous round of review and you feel that this manuscript is now acceptable for publication, you may indicate that here to bypass the "Comments to the Author" section, enter your conflict of interest statement in the "Confidential to Editor" section, and submit your "Accept" recommendation.
Reviewer #2: All comments have been addressed
Reviewer #3: (No Response)

2. Is the manuscript technically sound, and do the data support the conclusions?
The manuscript must describe a technically sound piece of scientific research with data that supports the conclusions. Experiments must have been conducted rigorously, with appropriate controls, replication, and sample sizes. The conclusions must be drawn appropriately based on the data presented.
Reviewer #2: Yes
Reviewer #3: Yes

3. Has the statistical analysis been performed appropriately and rigorously?
Reviewer #2: Yes
Reviewer #3: Yes

4. Have the authors made all data underlying the findings in their manuscript fully available?
The PLOS Data policy requires authors to make all data underlying the findings described in their manuscript fully available without restriction, with rare exception (please refer to the Data Availability Statement in the manuscript PDF file). The data should be provided as part of the manuscript or its supporting information, or deposited to a public repository. For example, in addition to summary statistics, the data points behind means, medians and variance measures should be available. If there are restrictions on publicly sharing data—e.g. participant privacy or use of data from a third party—those must be specified. Reviewer #2: Yes
Reviewer #3: Yes 5. Is the manuscript presented in an intelligible fashion and written in standard English?
PLOS ONE does not copyedit accepted manuscripts, so the language in submitted articles must be clear, correct, and unambiguous. Any typographical or grammatical errors should be corrected at revision, so please note any specific errors here.
Reviewer #2: Yes
Reviewer #3: Yes

6. Review Comments to the Author
Please use the space provided to explain your answers to the questions above. You may also include additional comments for the author, including concerns about dual publication, research ethics, or publication ethics. (Please upload your review as an attachment if it exceeds 20,000 characters)

Reviewer #2: This is my short review of the second revision of the manuscript "The evolution of scientific literature as metastable knowledge states". In my opinion, the authors adequately addressed the comments I (and other reviewers) left in previous review iterations. Overall, the manuscript looks good to me. Authors

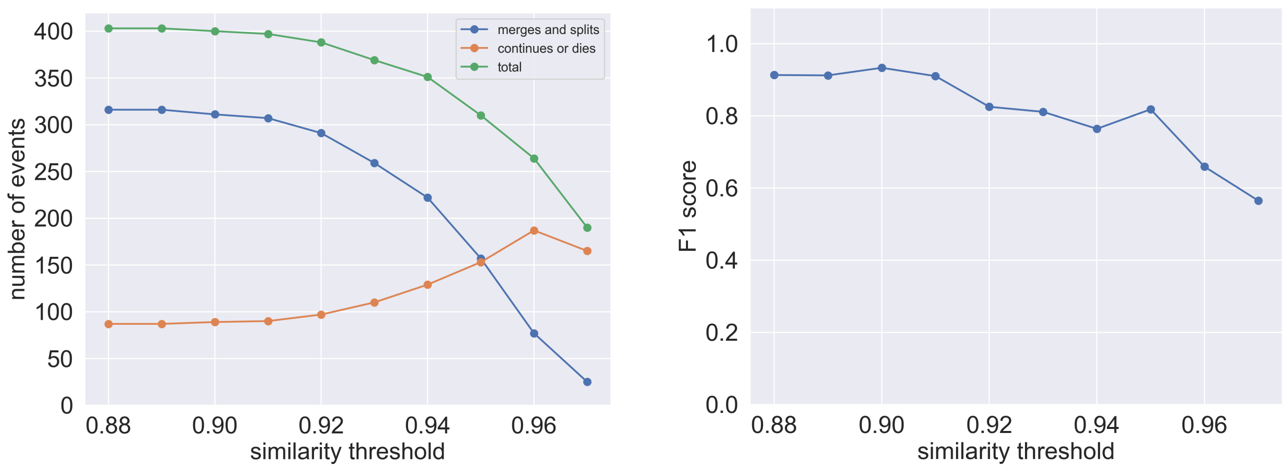

(a) fraction of event types vs threshold

(b) Predictive performance vs threshold

Figure 1: Figures showing the impact of similarity threshold value on fraction of different event groups identified as well as event prediction task performance.

explained their experiment design choices, limitations and proposed additional research directions in the "Discussion" section.

Reviewer #3: The authors use a large dataset to estimate how knowledge evolves over time. The paper has a good flow and is well argued. There is a necessarily complex data preparation and analysis given the size of the data and the many model choices. The authors gave thoughtful answers to my previous questions.

Some of the choices are arbitrary, for example matching clusters based on the cosine similarity of 0.95 or higher. Would things have been different if this had been 0.90 or 0.99?

To better understand the impact of threshold choice for cluster matching, we have carried out a series of experiments with varying thresholds. As one would expect, increasing the threshold (tightening the criteria for a cluster at time t to be considered the same as a cluster at time $t + 1$) leads to a decrease in the number of identified merges and splits. And consequently, an increase in the number of continuation and death events. Threshold values above 0.97 result in no merge events while lowering the threshold below 0.88 results in no continuation events. Figure 1 gives a summary of impacts of threshold choice on observed events. In this way, we can interpret the cluster similarity threshold as a parameter modulating selection from amongst a *hierarchical family of events* varying in resolution.

With respect to prediction task performance, performance is relatively stable for values between 0.88 and 0.91. However, increasing the threshold above 0.91 led to reduction in the performance in our dataset. We hypothesize this may be due to the reduction in number of events identified and thus less training.

We have added the full results of these additional experiments and discussion of these findings to the paper's Appendix and linked it in the paper (see Text-based dynamic event modeling).

It may be useful to do some random checks of the predictions to give confidence that the approach is working as expected. For example, randomly sampling papers and potential clusters, and asking blinded experts to classify the papers and then comparing this with the classifications made by the model.

We appreciate the suggestion. During this phase of revision, per the reviewer's recommendation, we asked blinded experts to classify papers from a randomly selected subset of our clusters and compare the results. Specifically, we asked three faculty members for input - one each from the fields of Marketing, Psychology, and Political Science. We asked them to complete two tasks.

Task 1: We showed each expert titles and abstracts corresponding with a collection of publications in their field (13 marketing papers, 12 each for psychology and political science). We asked them to group those papers into a fixed set of clusters (4 for marketing, 3 for psychology and political science).

Task 2: After they had completed their own clustering, we showed them the clusters our algorithm identified.

We asked them to rate the clusters using a Likert scale (1 to 5, where 1 represents *extremely bad* and 5 represents *extremely good*).
Results are summarized in Table 1. We use Jaccard similarity to score similarity between the expert-generated clusters and our clusters (Task 1). Despite variation in this outcome across the three fields, experts consistently gave high ratings when evaluating the clusters created by our model (Task 2). This appears to highlight the inherent challenge of coming up with a universal "ground truth" for defining similar papers or groups of papers in the literature.
Our survey also included an open field for any additional feedback. Following is the feedback we received:

Marketing: *One paper (13) is pure theory and more Econ but the others are data driven marketing papers*
Psychology: *The B category hangs well together with health and intervention work. It is the A and C groups that are more challenging as they cut across social and cognitive sciences. e.g., paper 6 is about collective cognition which should likely be paired with paper 12*
Political Science: *This was perfect – but imo this one was pretty easy, I could do the clustering through the titles alone*

Of note, the faculty member from Psychology defined clusters based on normative subfields within the discipline. Conversely, the faculty member from Marketing used differences in the theoretical vs. applied focus of the work as a primary criteria. These anecdotes offer insight into why previous work in science of science has generally relied on subject categories generated by sources like Web of Science as ground truth. However, we argue that existing categories are very high level and do not adequately account for the interdisciplinary nature of much of the literature. We have added to the paper a discussion of the outstanding need for benchmark datasets for taxonomization of interdisciplinary work (see Discussion).

Discipline	# clusters	# publications	Task 1: Mean Jaccard similarity	Task 2: Evaluation of algorithm-generated clusters (scale of 1-5)
Marketing	4	13	0.46	4
Psychology	3	12	0.7	4
Political Science	3	12	0.88	5

Table 1: Summary of results from a brief survey of three domain experts.
